# Characterization of *Anaplasma ovis* strains using the major surface protein 1a repeat sequences

**DOI:** 10.1186/s13071-017-2363-6

**Published:** 2017-09-29

**Authors:** Rong Han, Jifei Yang, Zhijie Liu, Shaodian Gao, Qingli Niu, Muhammad Adeel Hassan, Jianxun Luo, Hong Yin

**Affiliations:** 1 State Key Laboratory of Veterinary Etiological Biology, Key Laboratory of Veterinary Parasitology of Gansu Province, Lanzhou Veterinary Research Institute, Chinese Academy of Agricultural Sciences, Xujiaping 1, Lanzhou, Gansu 730046 People’s Republic of China; 2 Qinghai Provincial Center for Animal Disease Control and Prevention, Xining, 810003 Qinghai People’s Republic of China; 3 Jiangsu Co-innovation Center for Prevention and Control of Important Animal Infectious Diseases and Zoonoses, Yangzhou, 225009 People’s Republic of China

**Keywords:** *Anaplasma ovis*, *msp4* gene, Msp1a repeats, Genotypes, Sheep, Goats, China

## Abstract

**Background:**

*Anaplasma ovis* is one of the tick-transmitted pathogens of small ruminants. It causes ovine anaplasmosis and widely distributed in the world. In contrast to extensive worldwide genetic diversity of *A. marginale* and *A. phagocytophilum*, there are few reports on the classification of *A. ovis* strains. This study was conducted to investigate the occurrence and characterize *A. ovis* strains from goats and sheep from 12 provinces in China.

**Methods:**

The occurrence of *A. ovis* DNA was tested in 552 goats and sheep, by PCR based on the *msp4* gene. Positive samples were used for the amplification of the *msp1a* gene of *A. ovis*. The Msp1a amino acid repeats were further identified and used for the characterization of *A. ovis* strains.

**Results:**

The results showed that 79 (14.3%) goats and sheep were positive for *A. ovis*. The infection rates of *A. ovis* among different study sites ranged from 0 to 100%, and were significantly higher in sheep (26.6%, 45/169) than in goats (8.9%, 34/383) (*χ* ^2^ = 21.403, *df* = 1, *P* < 0.001). The *msp4* gene sequences of these isolates were 99.8–100% identical to each other, and they represented two sequence types. Forty-four partial *msp1a* gene sequences containing the repeat sequences were obtained from *A. ovis*-positive samples. After translation to amino acid sequences, 24 Msp1a repeats with 33 to 47 amino acids, which corresponded to 19 genotypes of *A. ovis*, were recognized in goats and sheep in China.

**Conclusions:**

*Anaplasma ovis* is widely distributed in the investigated geographical regions. The *msp4* gene of *A. ovis* had high sequence identity and was unable to be used to discriminate different strains. The Msp1a could be used as a genetic marker for characterizing *A. ovis*, and 19 genotypes of *A. ovis* were recognized in domestic small ruminants in China. The present study revealed, for the first time, the genetic diversity of *A. ovis* based on the analyses of Msp1a amino acid repeats.

## Background

*Anaplasma* are obligate intracellular Gram-negative rickettsial bacteria of medical and veterinary interest in both tropical and subtropical regions [1]. The disease caused by *Anaplasma* spp. has been recognized over a century, and is still an important issue worldwide [2, 3]. Since disclosure of zoonotic potential of *A. phagocytophilum* in 1994, there has been great interest in these bacteria [1, 4]. Until recently, six species have been recognized in the genus *Anaplasma*: *Anaplasma marginale*, *Anaplasma bovis*, *Anaplasma phagocytophilum*, *Anaplasma centrale* (*A. marginale centrale*), *Anaplasma platys* and *Anaplasma ovis* [5]. *Anaplasma carpa* has recently been described and considered as an emerging zoonotic pathogen in China [6]. The members in the genus *Anaplasma* differ in their cellular tropism, vectors, host range and pathogenicity [5].

Ovine anaplasmosis is caused by *A. ovis*, which is an obligate intra-erythrocytic pathogen of small ruminants [5, 7]. The causative agent was first described in sheep in 1912, and is widely distributed in Asia, Africa, Europe and the USA [7, 8]. This organism infects sheep, goats and some wild ruminants [9, 10]. Recently, an *A. ovis* variant was detected in a patient in Cyprus, indicated the zoonotic potential of this agent [11]. The life-cycle of *A. ovis* involves vertebrates and ticks, and animals can develop persistent infections and serve as reservoir hosts [12].

Currently, the identification and characterization of *A. ovis* mainly relies on the analysis of 16S rRNA and *msp4* genes; however, these genes are highly conserved among heterologous strains [3, 13]. In previous reports, the major surface protein 1a (Msp1a), encoded by the *msp1a* gene, has been recognized as a stable molecular marker for classifying strains of *A. marginale* [14]. It has been revealed that *A. marginale* Msp1a could have evolved on the strength of immune selection pressure and differs among strains due to variable sequences and numbers of tandem amino acid repeats located in the N-terminal region of the protein [15]. The repeated region of *A. marginale* Msp1a contains the adhesion domain for tick cells and erythrocytes, which is essential for the invasion and transmission of the organism [15]. Previous reports have reported that immunization of cattle with Msp1a induces partial protection when challenged with *A. marginale* [15, 16]. Recently, Msp1a has also been identified in *A. centrale*, although attempts on other *Anaplasma* species have been performed [17]. In this study, we investigated the occurrence of *A. ovis* in small domestic animals in China, and identified the *msp1a* gene from *A. ovis*-positive samples. The *A. ovis* isolates identified herein were subsequently characterized based on the Msp1a amino acid repeats.

## Methods

### Sample collection and DNA preparation

Blood samples were obtained from March to September between 2011 and 2015 in 24 counties from 12 provinces of China (Table 1). Five hundred and fifty-two asymptomatic small ruminants (sheep, *n* = 169; goats, *n* = 383) were randomly selected in two to three sampling sites from each county included in this study. Blood samples were collected from the jugular vein of individual animals and collected in a sterile 10 ml vacutainer EDTA tubes and stored at 4 °C. DNA was prepared from 300 μl of blood by using the Gentra Puregene Blood Kit (Qiagen, Beijing, China) following the manufacturer’s instructions.

**Table 1:** Prevalence of *A. ovis* in goats and sheep from China, 2011–2015

Location		Species	No. infected	
Province	Country		No. tested	No. positive (%)
Chongqing	Wanzhou	Goat	24	0 (0)
	Jiangjin	Goat	30	0 (0)
Guangxi	Pingxiang	Goat	11	0 (0)
	Jingxi	Goat	19	0 (0)
Guizhou	Dushan	Goat	17	4 (23.5)
	Rongjiang	Goat	29	1 (3.4)
Hebei	Baoding	Sheep	19	0 (0)
Liaoning	Haicheng	Goat	23	1 (4.3)
	Huangren	Goat	16	0 (0)
	Fengcheng	Goat	14	0 (0)
Hainan	Haikou	Goat	28	6 (21.4)
Inner Mongolia	Manzhouli	Sheep	13	0 (0)
	Xinbaerhuzuoqi	Sheep	20	14 (70.0)
	Aershan	Sheep	20	4 (20.0)
	Eerguna	Goat	20	5 (25.0)
Sichuan	Hejiang	Goat	32	0 (0)
	Panzhihua	Goat	31	13 (41.9)
Shanxi	Lvliang	Sheep	50	22(44.0)
Guangdong	Qingyuan	Goat	30	0 (0)
	Zhaoqing	Goat	33	0 (0)
Yunnan	Ruili	Goat	4	4 (100)
	Fuyuan	Goat	7	0 (0)
	Yanshan	Goat	15	0 (0)
Hubei	Suizhou	Sheep	47	5 (10.6)
Total			552	79 (14.3)

### PCR reactions

Specific DNA of *A. ovis* was detected by PCR based on *msp4* gene with primer set MSP45 (5′-GGG AGC TCC TAT GAA TTA CAG AGA ATT GTT TAC-3′) and MSP43 (5′-CCG GAT CCT TAG CTG AAC AGA ATC TTG C-3′) as described previously, which generated a product of 869 bp [13]. The DNA of *A. ovis* strain Haibei (GenBank accession no. GQ483471) and sterile water were used as the positive and negative control, respectively. Amplification products were analyzed by 1.0% agarose gel electrophoresis. The *msp1a* gene was further amplified from *A. ovis*-positive samples. Primers AoMsp1aF (5′-CGT TTC CAT GTG CTA CAA TGC CG-3′) and AoMsp1aR (5′-GCT GTT CGC TAT CGC AGT CTG TG-3′) were designed based on the *A. ovis* strain Haibei genome sequence (GenBank accession no. CP007596, unreleased) to target repeat sequences within the *msp1a* gene. The PCR reaction system is consistent with the amplification of *msp4* gene. Thermal cycling conditions include 94 °C for 4 min, 35 cycles at 94 °C for 30 s, 55 °C for 30 s, and 72 °C for 30 s, with a final extension step at 72 °C for 5 min.

### Sequence and statistical analysis

The amplified fragments of *msp4* and *msp1a* genes were purified and cloned into pGEM-T Easy vector (Promega, Madison, WI, USA). At least two recombinants were sequenced from each amplification (Genscript, Nanjing, China). The *msp4* gene sequences have been deposited in GenBank (accession numbers KY807127 and KY807128) and were analyzed by the BLASTn search and the ClustalW software (DNAStar, Madison, WI, USA). The *msp1a* gene sequences were edited and translated to amino acids by using CLC Genomics Workbench 7.5.1 (Qiagen, Aarhus, Denmark). The amino acid repeat sequences were identified and named Ao*n*. These repeats were aligned using the ClustalV method in the MegAlign software. Statistical analysis was performed with a Chi-square test in Predictive for Analytics Software Statistics 18 (PASW, SPSS Inc., Chicago, IL, USA), and a difference was considered statistically significant at *P <* 0.05.

## Results

In total, 552 blood samples from goats and sheep were screened for the presence of *msp4* gene of *A. ovis*. The results showed that 79 (14.3%) sampled animals were positive for *A. ovis* (Table 1). The prevalence of *A. ovis* among different study regions ranged between 0 and 100%, and were significantly higher in sheep (26.6%, 45/169) than in goats (8.9%, 34/383) (*χ* ^2^ = 21.403, *df* = 1, *P* < 0.001) (Table 1).

The *A. ovis* infections in goats and sheep were further confirmed by sequencing, and 42 *msp4* gene sequences were obtained. The *msp4* gene sequences shared 99.8–100% similarities, and they represented two sequence types. Eighteen *msp4* sequences (13 from sheep and 5 from goats, GenBank accession no. KY807127) were identical to the strains Italy 147 and Yuzhong of *A. ovis*, which were detected in sheep from Italy and China (GenBank: AY702924 and HQ456348, respectively) [18, 19]. The remaining 24 *msp4* sequences (8 from sheep and 16 from goats, GenBank: KY807127) have 99.9% identity to the *A. ovis* strains ATS20, Yongjing and Italy 20 derived from sheep (GenBank: KJ782397, HQ456347 and AY702923) [18–20].

**Fig. 1:**
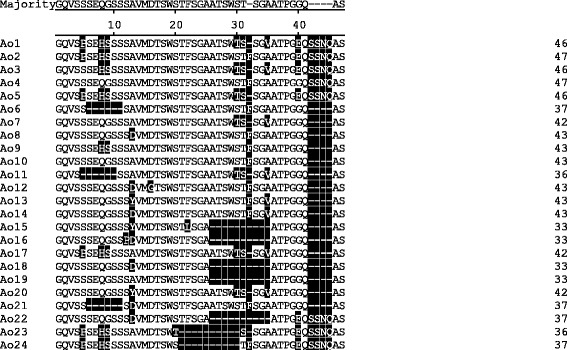
The Msp1a amino acid repeat sequences of *A. ovis* geographical strains identified from goats and sheep. The ID of each repeat type was named as Ao1–24, which were aligned using the ClustalV method in the MegAlign software. The one letter code was used to reveal the different amino acid sequences of Msp1a repeats. The variable amino acids are highlighted on a black background and gaps indicate deletions/insertions

Forty-four partial *msp1a* gene sequences contained the repeat sequences were obtained from *A. ovis*-positive samples. After translated to amino acid sequences, 24 different types of Msp1a repeats of *A. ovis* were identified and named Ao1–24 in this study (Fig. 1, partial *msp1a* amino acid sequences are available in Additional file 1: Table S1). These Msp1a repeats were highly variable with 33 to 47 amino acids, and several positions (GQVS---------VM-TSW----------------ATPG-Q---QAS) were totally conserved (Fig. 1).

The structure of the Msp1a repeats region was represented using the amino acid repeat types for isolates of *A. ovis*. Overall, 44 isolates of *A. ovis* were classified and resulted in 19 genotypes based on the organization of different amino acid repeats (Table 2). Aside from one isolate (A18-18a, Ao18/Ao19/Ao11) that had three amino acid repeats, the remaining 43 isolates contained two amino acid repeats (Table 2). Five of 24 Msp1a repeat sequences (Ao5, Ao6, Ao8, Ao10 and Ao11) were shared between different isolates. The repeat Ao6 was the most common repeat sequence, occurring in eight genotypes of 27 isolates (Table 2). However, most of the repeats had a low frequency, one time in only one strain (Table 2). According to the organization of Msp1a repeats in *A. ovis* isolates, ten genotypes (Ao1/Ao6, Ao2/Ao6, Ao3/Ao6, Ao5/Ao6, Ao7/Ao6, Ao10/Ao8, Ao15/Ao16, Ao22/Ao6, Ao23/Ao10, and Ao24/Ao5) were identified in goats and nine genotypes (Ao4/Ao6, Ao4/Ao11, Ao9/Ao10, Ao10/Ao13, Ao12/Ao8, Ao14/Ao8, Ao17/Ao6, Ao18/Ao19/Ao11, and Ao20/Ao21) were found in sheep.

**Table 2:** Organization of Msp1a repeats in *A. ovis* strains identified in goats and sheep. The structure of the Msp1a repeats region was represented using the repeat types showed in Fig. 1 for strains of *A. ovis*

*A. ovis* strains	Origin	Host	Structure of Msp1a repeats		
A7-1b, A7-12c, A7-20a, A7-20b	Hainan	Goat	Ao1	Ao6	
A22-2a, A22-2b, A22-7b	Yunnan	Goat	Ao1	Ao6	
DSS5C, DSS16A, DSS16B	Guizhou	Goat	Ao1	Ao6	
PZH41A, PZH41C	Sichuan	Goat	Ao1	Ao6	
A22-7a	Yunnan	Goat	Ao2	Ao6	
PZH46B, PZH46C, PZH60B, PZH60C	Sichuan	Goat	Ao2	Ao6	
A7-1c	Hainan	Goat	Ao3	Ao6	
PZH60A	Sichuan	Goat	Ao3	Ao6	
A19-17a	Inner Mongolia	Sheep	Ao4	Ao6	
A19-17b	Inner Mongolia	Sheep	Ao4	Ao11	
DSS16C, DSS16D	Guizhou	Goat	Ao5	Ao6	
A22-3a	Yunnan	Goat	Ao5	Ao6	
A7-16a, A7-16b	Hainan	Goat	Ao7	Ao6	
A18-32b, A18-32c	Shanxi	Sheep	Ao9	Ao10	
A7-17a, A7-17b	Hainan	Goat	Ao10	Ao8	
A18-3b, A18-6a, A18-6c	Shanxi	Sheep	Ao10	Ao13	
A19-12a, A19-12b	Inner Mongolia	Sheep	Ao12	Ao8	
A19-1a, A19-1b	Inner Mongolia	Sheep	Ao14	Ao8	
A8-105b	Inner Mongolia	Goat	Ao15	Ao16	
A18-7b	Shanxi	Sheep	Ao17	Ao6	
A18-18a	Shanxi	Sheep	Ao18	Ao19	Ao11
A18-32a	Shanxi	Sheep	Ao20	Ao21	
A22-3b	Yunnan	Goat	Ao22	Ao6	
DSS25B	Guizhou	Goat	Ao23	Ao10	
PZH41B	Sichuan	Goat	Ao24	Ao5	

## Discussion

Ovine anaplasmosis is widely distributed and causes mild clinical symptoms [21]. *Anaplasma ovis* was first described in sheep as early as 1982 in Xinjiang Uygur Autonomous Region, and it was subsequently detected in goats in Liaoning province in China [22]. After that, several molecular epidemiological investigations of *A. ovis* have been conducted in domestic and wild ruminants from different geographical locations [23]. In those reports, *A. ovis* was found in 88 of 621 sheep (14.2%) and in 129 of 710 goats (18.2%) from six provinces [24]; in 51 of 125 sheep (40.5%) from Xinjiang [20]; and in goats from Henan (8.7%), Hubei (7.2%), Guizhou (17.8%) and Zhejiang (26.3%), with an overall prevalence of 15.3% (40/262) [25]. Apart from domestic ruminants, *A. ovis* has also been found in mongolian gazelle (*Procapra gutturosa*) (48/92, 52.2%) [10], red deer (*Cervus elaphus*) (14/44, 32.0%), sika deer (*Cervus nippon nippon*) (8/40, 20.0%) [9], and dogs (6.1%, 15/243) [26]. Moreover, the DNA of *A. ovis* has been detected in milk samples from goats and sheep in China [27]. In this study, *A. ovis* was detected in 79/552 (14.3%) goats and sheep, and it was found in 11 of 24 counties studied. The positive rates of *A. ovis* were variable in goats and sheep, as well as between different geographical locations. These findings revealed that *A. ovis* is widely distributed in the sites investigated, implying that ovine anaplasmosis caused by *A. ovis* appears to be frequent in China.

Molecular characterization of *Anaplasma* has relied mainly on analyses of various gene loci [3]. The target genes used to determine the genetic diversity of *A. ovis* include the *16S* rRNA and *msp4* genes, and several genotypes and genetic variants have been identified in previous reports [22, 25, 28–32]. However, these molecular markers were found to be highly conserved and not informative enough to delineate *A. ovis* isolates [3, 13, 22]. In this study, we also found that the *msp4* gene of *A. ovis* isolates identified from goats and sheep shared high sequence similarity (99.8–100%), and were unable to reveal the genetic characterization of these isolates.

The major surface proteins of the members in the genus *Anaplasma* have been well characterized, especially in *A. marginale* and *A. phagocytophilum* [33, 34]. The Msp1a has been extensively used as a molecular marker for characterizing *A. marginale* strains on the basis of the variable N-terminal region, containing the repeated peptides [15]. To date, over 200 *A. marginale* Msp1a tandem repeats have been identified, and a great number of strains from different countries have been classified into a variety of genotypes [3, 15, 17]. In this study, we examined *A. ovis*-positive samples for Msp1a genotype, and 24 Msp1a repeats with 33–47 amino acids, which corresponded to 19 *A. ovis* genotypes identified in goats and sheep in China. The structure of Msp1a tandem repeat and the amino acid sequences vary among strains, which has also been shown for *A. marginale*.

It has been reported that the Msp1a of *A. marginale* interact with vertebrate host and tick cells and have evolved on the strength of immune pressure [15]. This study revealed high genetic diversity of *A. ovis* isolates in small domestic ruminants in China, suggesting that *msp1a* gene of *A. ovis* may also have evolved more obviously than other genes. The *A. ovis* strains identified in this study had two to three Msp1a repeats, some of which were shared between different strains. However, no significant association was observed between specific tandem repeats and host or geographical regions in this study, since some repeats were identified in both goats and sheep and distributed extensively (repeat Ao6, Ao8 and Ao10 identified in goats and sheep from several provinces). Moreover, same genotypes of *A. ovis* were found in several provinces (Ao1/Ao6, Ao2/Ao6, etc.); this may be attributed to the animal movement between those provinces.

To date, characterizing *A. marginale* strains based on MSP1a repeat sequences has been well studied. The present study, for the first time, revealed the genetic diversity of *A. ovis* using Msp1a repeats in goats and sheep in China. Due to the wide distribution of *A. ovis*, more studies should be conducted in vertebrate and invertebrate hosts from different countries, which will ultimately provide more evolutionary and phylogenetic information about *A. ovis* strains.

## Conclusions

*Anaplasma ovis* was molecularly detected in goats and sheep from 12 provinces in China, with an overall infection rate of 14.3%. The *msp4* gene of *A. ovis* had high sequence identity and was unable to be used to discriminate different strains. The Msp1a could be used as a genetic marker for characterizing *A. ovis*, and 24 Msp1a repeats with 33–47 amino acids, which corresponded to 19 genotypes of *A. ovis*, were identified in goats and sheep in China. The present study provided the first evidence of genetic diversity of *A. ovis* based on the analyses of Msp1a repeats.

## Additional file

Additional file 1: Table S1. Partial *msp1a* amino-acid sequences containing repeat sequences of *A. ovis* strains analyzed in this study. (DOCX 31 kb)

## Abbreviations

Msp: Major surface protein
UV: ultraviolet
