# Supplementary material for: Characterization of Anaplasma ovis strains using the major surface protein 1a repeat sequences
Source: Parasit Vectors. 2017 Sep 29;10:447. doi: 10.1186/s13071-017-2363-6 (PMC5622584; doi:10.1186/s13071-017-2363-6)
Supplement: Additional file 1: Table S1. — Partial msp1a amino-acid sequences containing repeat sequences of A. ovis strains analyzed in this study. (DOCX 31 kb) [file 13071_2017_2363_MOESM1_ESM.docx]

**Additional file 1: Table S1.** Partial *msp1a* amino acid sequences containing repeat sequences of *A. ovis* strains analyzed in the present study.

>A7-1b

MSDNNQGQVSPSEHSSSSAVMDTSWSTFSGAATSWTSSGVATPGEQSSNQASQPPVEQQEGSQDQGQVSSSAVMDTSWSTFSGAATSWSTFSGAATPGGQASGGTVDWQEAISDKIGSREYMDAARSI

>A7-1c

MSDNNQGQVSSSEHSSSSAVMDTSWSTFSGAATSWTSSGVATPGEQSSNQASQPPVEQQEGSQDQGQVSSSAVMDTSWSTFSGAATSWSTFSGAATPGGQASGGTVDWQEAISDKIGSREYMDAARSI

>A7-12c

MSDNNQGQVSPSEHSSSSAVMDTSWSTFSGAATSWTSSGVATPGEQSSNQASQPPVEQQEGSQDQGQVSSSAVMDTSWSTFSGAATSWSTFSGAATPGGQASGGTVDWQEAISDKIGSREYMDAARSI

>A7-16a

MSDNNQGQVSSSEQGSSSAVMDTSWSTFSGAATSWTSSGVATPGGQASNQEASQSPVEQQEGSQDQGQVSSSAVMDTSWSTFSGAATSWSTFSGAATPGGQASGGTVDWQKAISDKIGSQEYMDAARSIIAVGIYA

>A7-16b

MSDNNQGQVSSSEQGSSSAVMDTSWSTFSGAATSWTSSGVATPGGQASNQEASQSPVEQQEGSQDQGQVSSSAVMDTSWSTFSGAATSWSTFSGAATPGGQASGGTVDWQKAISDKIGSQEYMDAARSIIAVGIYA

>A7-17a

MSDNNQGQVSSSEQGSSSAVMDTSWSTFSGAATSWSTFSGAATPGGQASNQASQPPVERQEESQGQVSSSEQGSSSDVMDTSWSTFSGAATSWSTFSGAATPGGQASGGTVDWQKAISDKIGSQEYMDAARSIIAVGIYA

>A7-17b

MSDNNQGQVSSSEQGSSSAVMDTSWSTFSGAATSWSTFSGAATPGGQASNQASQPPVERQEESQGQVSSSEQGSSSDVMDTSWSTFSGAATSWSTFSGAATPGGQASGGTVDWQKAISDKIGSQEYMDAARSIIAVGIYA

>A7-20a

MSDNNQGQVSPSEHSSSSAVMDTSWSTFSGAATSWTSSGVATPGEQSSNQASQPPVEQQEGSQDQGQVSSSAVMDTSWSTFSGAATSWSTFSGAATPGGQASGGTVDWQEAISDKIGSREYMDAARSIIAVGIYA

>A7-20b

MSDNNQGQVSPSEHSSSSAVMDTSWSTFSGAATSWTSSGVATPGEQSSNQASQPPVEQQEGSQDQGQVSSSAVMDTSWSTFSGAATSWSTFSGAATPGGQASGGTVDWQEAISDKIGSREYMDAARSIIAVGIYA

>A8-105b

MSDNNQGQVSSSEQGSSSYVMDTSWSTLSGAATPGGQASNQEASQSPVEQQEGSQGQVSSSGQVSSSEQGSSPDVMDTSWSTFSGAATPGGQASGGTVDWQKAISDKIGSQEYMDAARSI

>A18-3b

MSDNNQGQVSSSEQGSSSAVMDTSWSTFSGAATSWSTFSGAATPGGQASNQEASQSPVEQQGGSQDQVSSSGQVSSSEQGSSSYVMDTSWSTFSGAATSWSTFSGVATPGGQASGGTVDWQEAISDKIGSQEYMDAARSI

>A18-6a

MSDNNQGQVSSSEQGSSSAVMDTSWSTFSGAATSWSTFSGAATPGGQASNQEASQSPVEQQGGSQDQVSSSGQVSSSEQGSSSYVMDTSWSTFSGAATSWSTFSGVATPGGQASGGTVDWQEAISDKIGSQEYMDAARSI

>A18-6c

MSDNNQGQVSSSEQGSSSAVMDTSWSTFSGAATSWSTFSGAATPGGQASNQEASQSPVEQQGGSQDQVSSSGQVSSSEQGSSSYVMDTSWSTFSGAATSWSTFSGVATPGGQASGGTVDWQEAISDKIGSQEYMDAARSI

>A18-7b

MSDNNQGQVSPSEHSSSSAVMDTSWSTFSGAATSWTSSGVATPGGQASNQEASQSPVEQQGGSQDQGQVSSSAVMDTSWSTFSGAATSWSTFSGAATPGGQASGGTVDWQKAISDKIGSQEYMDAARSI

>A18-18a

MSDNNQGQVSSSEQGSSSDVMDTSWSTFSGAATPGGQASNQEASQSPVEQQGGSQDQVSSSGQVSSSEQGSSSAVMDTSWSTFSGAATPGGQASNQEASQSPVEQQEGSRDQGQVSSSAVMDTSWSTFSGAATSWTSSGVATPGGQASGGTVDWQEAISDKIGSREYMDAARSI

>A18-32a

MSDNNQGQVSSSEQGSSSYVMDTSWSTFSGAATSWTSSGVATPGGQASNQEASQSPVEQQEGSQDQGQVSSSDVMDTSWSTFSGAATSWSTFSGAATPGGQASGGTVDWQKAISDKIGSQEYMDAARSIIAVGIYA

>A18-32b

MSDNNQGQVSSSEHSSSSAVMDTSWSTFSGAATSWSTFSGAATPGGQASNQEASQSPVEQQEGSQDQVSSSGQVSSSEQGSSSAVMDTSWSTFSGAATSWSTFSGAATPGGQASGGTVDWQEAISDKIGSQEYMDAARSIIAVGIYA

>A18-32c

MSDNNQGQVSSSEHSSSSAVMDTSWSTFSGAATSWSTFSGAATPGGQASNQEASQSPVEQQEGSQDQVSSSGQVSSSEQGSSSAVMDTSWSTFSGAATSWSTFSGAATPGGQASGGTVDWQEAISDKIGSQEYMDAARSIIAVGIYA

>A19-1a

MSDNNQGQVSSSEQGSSSDVMDTSWSTFSGAATSWSTFSGVATPGGQASNQEAGQSPVEQQGGSQDQVSSSGQVSSSEQGSSSDVMDTSWSTFSGAATSWSTFSGAATPGGQASGGTVDWQEAISDKIGSREYMDAARSIIAVGIYA

>A19-1b

MSDNNQGQVSSSEQGSSSDVMDTSWSTFSGAATSWSTFSGVATPGGQASNQEAGQSPVEQQGGSQDQVSSSGQVSSSEQGSSSDVMDTSWSTFSGAATSWSTFSGAATPGGQASGGTVDWQEAISDKIGSREYMDAARSIIAVGIYA

>A19-12a

MSDNNQGQVSSSEQGSSSDVMGTSWSTFSGAATSWSTFSGAATPGGQASNQEASQSPVEQQGGSQDQVSSSGQVSSSEQGSSSDVMDTSWSTFSGAATSWSTFSGAATPGGQASGGTVDWQKAISDKIGSQEYMDAARSIIAVGIYA

>A19-12b

MSDNNQGQVSSSEQGSSSDVMGTSWSTFSGAATSWSTFSGAATPGGQASNQEASQSPVEQQGGSQDQVSSSGQVSSSEQGSSSDVMDTSWSTFSGAATSWSTFSGAATPGGQASGGTVDWQKAISDKIGSQEYMDAARSIIAVGIYA

>A19-17a

MSDNNQGQVSSSEQGSSSAVMDTSWSTFSGAATSWSTFSGAATPGGQSSNQASQPPVEQQGGSQDQGQVSSSAVMDTSWSTFSGAATSWSTFSGAATPGGQASGGTVDWQEAISDKIGSQEYMDAARSIIAVGIYA

>A19-17b

MSDNNQGQVSSSEQGSSSAVMDTSWSTFSGAATSWSTFSGAATPGGQSSNQASQPPVEQQGGSQDQGQVSSSAVMDTSWSTFSGAATSWTSSGVATPGGQASGGTVDWQEAISDKIGSQEYMDAARSIIAVGIYA

>A22-2a

MSDNNQGQVSPSEHSSSSAVMDTSWSTFSGAATSWTSSGVATPGEQSSNQASQPPVEQQEGSQDQGQVSSSAVMDTSWSTFSGAATSWSTFSGAATPGGQASGGTVDWQEAISDKIGSREYMDAARSI

>A22-2b

MSDNNQGQVSPSEHSSSSAVMDTSWSTFSGAATSWTSSGVATPGEQSSNQASQPPVEQQEGSQDQGQVSSSAVMDTSWSTFSGAATSWSTFSGAATPGGQASGGTVDWQEAISDKIGSREYMDAARSI

>A22-3a

MSDNNQGQVSPSEHSSSSAVMDTSWSTFSGAATSWTSSGAATPGEQSSNQASQPPVEQQEGSQDQGQVSSSAVMDTSWSTFSGAATSWSTFSGAATPGGQASGGTVDWQEAISDKIGSREYMDAARSIIAVGIYA

>A22-3b

MSDNNQGQVSSSEQGSSSDVMDTSWSTFSGAATPGEQSSNQASQPPVEQQEGSQDQGQVSSSAVMDTSWSTFSGAATSWSTFSGAATPGGQASGGTVDWQEAISDKIGSREYMDAARSIIAVGIYA

>A22-7a

MSDNNQGQVSPSEHSSSSAVMDTSWSTFSGAATSWSTFSGAATPGEQSSNQASQPPVEQQEGSQDQGQVSSSAVMDTSWSTFSGAATSWSTFSGAATPGGQASGGTVDWQEAISDKIGSREYMDAARSI

>A22-7b

MSDNNQGQVSPSEHSSSSAVMDTSWSTFSGAATSWTSSGVATPGEQSSNQASQPPVEQQEGSQDQGQVSSSAVMDTSWSTFSGAATSWSTFSGAATPGGQASGGTVDWQEAISDKIGSREYMDAARSIIAVGIYA

>DSS5C

MSDNNQGQVSPSEHSSSSAVMDTSWSTFSGAATSWTSSGVATPGEQSSNQASQPPVEQQEGSQDQGQVSSSAVMDTSWSTFSGAATSWSTFSGAATPGGQASGGTVDWQEAISDKIGSREYMDAARSIIAVGIYA

>DSS16A

MSDNNQGQVSPSEHSSSSAVMDTSWSTFSGAATSWTSSGVATPGEQSSNQASQPPVEQQEGSQDQGQVSSSAVMDTSWSTFSGAATSWSTFSGAATPGGQASGGTVDWQEAISDKIGSREYMDAARSIIAVGIY

>DSS16B

MSDNNQGQVSPSEHSSSSAVMDTSWSTFSGAATSWTSSGVATPGEQSSNQASQPPVEQQEGSQDQGQVSSSAVMDTSWSTFSGAATSWSTFSGAATPGGQASGGTVDWQEAISDKIGSREYMDAARSIIAVGIY

>DSS16C

MSDNNQGQVSPSEHSSSSAVMDTSWSTFSGAATSWTSSGAATPGEQSSNQASQPPVEQQEGSQDQGQVSSSAVMDTSWSTFSGAATSWSTFSGAATPGGQASGGTVDWQEAISDKIGSREYMDAARSIIAVGIYA

>DSS16D

MSDNNQGQVSPSEHSSSSAVMDTSWSTFSGAATSWTSSGAATPGEQSSNQASQPPVEQQEGSQDQGQVSSSAVMDTSWSTFSGAATSWSTFSGAATPGGQASGGTVDWQEAISDKIGSREYMDAARSIIAVGIYA

>DSS25B

MSDNNQGQVSPSEHSSSSAVMDTSWTSSGAATPGEQSSNQASQPPVEQQGGSQDQVSSSGQVSSSEQGSSSAVMDTSWSTFSGAATSWSTFSGAATPGGQASGGTVDWQEAISDKIGSREYMDAARSIIAVGIY

>PZH41A

MSDNNQGQVSPSEHSSSSAVMDTSWSTFSGAATSWTSSGVATPGEQSSNQASQPPVEQQEGSQDQGQVSSSAVMDTSWSTFSGAATSWSTFSGAATPGGQASGGTVDWQEAISDKIGSREYMDAARSIIAVGIYA

>PZH41C

MSDNNQGQVSPSEHSSSSAVMDTSWSTFSGAATSWTSSGVATPGEQSSNQASQPPVEQQEGSQDQGQVSSSAVMDTSWSTFSGAATSWSTFSGAATPGGQASGGTVDWQEAISDKIGSREYMDAARSIIAVGIY

>PZH41B

MSDNNQGQVSPSEHSSSSAVMDTSWSTFSGAATPGEQSSNQASQPPVEQQGGSQGQVSPSEHSSSSAVMDTSWSTFSGAATSWTSSGAATPGEQSSNQASQPPVEQQEGSQDQGQVSSSAVMDTSWSTFSG

>PZH46B

MSDNNQGQVSPSEHSSSSAVMDTSWSTFSGAATSWSTFSGAATPGEQSSNQASQPPVEQQEGSQDQGQVSSSAVMDTSWSTFSGAATSWSTFSGAATPGGQASGGTVDWQEAISDKIGSREYMDAARSIIAVGIYA

>PZH46C

MSDNNQGQVSPSEHSSSSAVMDTSWSTFSGAATSWSTFSGAATPGEQSSNQASQPPVEQQEGSQDQGQVSSSAVMDTSWSTFSGAATSWSTFSGAATPGGQASGGTVDWQEAISDKIGSREYMDAARSIIAVGIY

>PZH60A

MSDNNQGQVSSSEHSSSSAVMDTSWSTFSGAATSWTSSGVATPGEQSSNQASQPPVEQQEGSQDQGQVSSSAVMDTSWSTFSGAATSWSTFSGAATPGGQASGGTVDWQEAISDKIGSREYMDAARSIIAVGIY

>PZH60B

MSDNNQGQVSPSEHSSSSAVMDTSWSTFSGAATSWSTFSGAATPGEQSSNQASQPPVEQQEGSQDQGQVSSSAVMDTSWSTFSGAATSWSTFSGAATPGGQASGGTVDWQEAISDKIGSREYMDAARSVIAVGIYA

>PZH60C

MSDNNQGQVSPSEHSSSSAVMDTSWSTFSGAATSWSTFSGAATPGEQSSNQASQPPVEQQEGSQDQGQVSSSAVMDTSWSTFSGAATSWSTFSGAATPGGQASGGTVDWQEAISDKIGSREYMDAARSVIAVGIYA
